# Inequitable access to substance abuse treatment services in Cape Town, South Africa

**DOI:** 10.1186/1747-597X-5-28

**Published:** 2010-11-15

**Authors:** Bronwyn J Myers, Johann Louw, Sonja C Pasche

**Affiliations:** 1 Alcohol and Drug Abuse Research Unit, South African Medical Research Council, PO Box 19070, Tygerberg, 7505, South Africa; 2 Department of Psychiatry and Mental Health, University of Cape Town, Private Bag, Rondebosch, 7705, South Africa; 3 Department of Psychology, University of Cape Town, Private Bag, Rondebosch, 7705, South Africa

## Abstract

**Background:**

Despite high levels of substance use disorders in Cape Town, substance abuse treatment utilization is low among people from disadvantaged communities in Cape Town, South Africa. To improve substance abuse treatment utilization, it is important to identify any potential barriers to treatment initiation so that interventions to reduce these barriers can be implemented. To date, substance abuse research has not examined the factors associated with substance abuse treatment utilization within developing countries. Using the Behavioural Model of Health Services Utilization as an analytic framework, this study aimed to redress this gap by examining whether access to substance abuse treatment is equitable and the profile of variables associated with treatment utilization for people from poor communities in Cape Town, South Africa.

**Methods:**

This study used a case-control design to compare 434 individuals with substance use disorders from disadvantaged communities who had accessed treatment with 555 controls who had not accessed treatment on a range of predisposing, treatment need and enabling/restricting variables thought to be associated with treatment utilization. A hierarchical logistic regression was conducted to assess the unique contribution that the need for treatment, predisposing and enabling/restricting variable blocks made on substance abuse treatment utilization.

**Results:**

Findings revealed that non-need enabling/restricting variables accounted for almost equal proportions of the variance in service utilization as the need for treatment variables. These enabling/restricting variables also attenuated the influence of the treatment need and predisposing variables domains on chances of treatment utilization. Several enabling/restricting variables emerged as powerful partial predictors of utilization including competing financial priorities, geographic access barriers and awareness of treatment services. Perceived severity of drug use, a need for treatment variable) was also a partial predictor of utilization.

**Conclusions:**

Findings point to inequitable access to substance abuse treatment services among people from poor South African communities, with non-need factors being significant determinants of treatment utilization. In these communities, treatment utilization can be enhanced by (i) expanding the existing repertoire of services to include low threshold services that target individuals with less severe problems; (ii) providing food and transport vouchers as part of contingency management efforts, thereby reducing some of the financial and geographic access barriers; (iii) introducing community-based mobile outpatient treatment services that are geographically accessible; and (iv) employing community-based outreach workers that focus on improving awareness of where, when and how to access existing treatment services.

## Background

Findings from national epidemiological research point to high rates of untreated substance use disorders in South Africa [1,2], with one study reporting that 13% of the general population had a current (untreated) substance use disorder [1]. The need to respond to these problems is apparent from the large burden these problems place, when untreated, on health care and legal systems in South Africa particularly in the Western Cape region [3,4]. Compared to the other provinces in the country, the Western Cape has significantly higher rates of substance-related problems [1]. Cape Town, the capital of the Western Cape, is particularly affected by these problems, with higher proportions of arrestees [3] and trauma patients [4] testing positive for alcohol and other drugs than other major cities in the country. Taken together, these findings underline the need for accessible substance abuse treatment services in Cape Town.

Although there is a need for substance abuse treatment services, access to treatment remains limited in Cape Town [5,6]. In part, this is due to the limited availability of services [6], with existing services only able to treat approximately 3500 persons per year [7]. This is grossly inadequate in a city with an estimated 15 000 heroin users [8], and in which approximately 10% of a population of 3 million people meet DSM-IV-R criteria for alcohol abuse and/or dependence [2]. Even within the context of limited service availability, the utilization of substance abuse treatment services in Cape Town has decreased over time, from a high of 3058 slots in the second half of 2007 to 2642 slots in the second half of 2009 [7]. As no substance abuse treatment facilities closed during this period, it seems that service availability is not the only factor informing the likelihood of people utilizing substance abuse treatment in Cape Town.

While the limited number of treatment slots available reduces the chances of accessing treatment for all South Africans, these services are relatively more difficult to access for people from Black/African and Coloured communities disadvantaged during the course of apartheid compared to their White counterparts [5]. The terms "Black/African" and "Coloured" refer to demographic markers used in South Africa. These markers are important as accurate user profiles assist in identifying vulnerable population subgroups and in planning effective intervention programmes. Many of the service delivery inequities associated with apartheid still need to be redressed in democratic South Africa, with large socio-economic inequities existing between Black and White South Africans [8]. To redress this situation and improve substance abuse treatment utilization among people from disadvantaged communities, it is important to identify the profile of factors associated with substance abuse treatment initiation so that any barriers to treatment utilization can be addressed.

Studies conducted in the USA have identified several factors positively associated with substance abuse treatment utilization, including perceived need for treatment, problem severity [9,10], psychological functioning [11] and social support for abstinence [12]. Factors shown to restrict treatment utilization include affordability barriers, limited awareness about where to seek help, geographic access barriers, stigma [9,11,13-15] and neighbourhood environment [16,17]. In the USA, need for treatment, as indicated by problem severity and perceived need for services, is often the factor most strongly associated with service utilization [9,10]. However, the extent to which these findings are applicable to the South African context is unknown. This is largely because treatment services research in South Africa has not compared recipients of services with community-based samples of untreated persons. This has made it difficult to identify factors that facilitate or restrict substance abuse treatment utilization for people from disadvantaged communities and has hampered the development of interventions to improve service utilization.

To redress this gap, we employed the Behavioural Model of Health Services Utilization (BHSU) [18], which served as the theoretical basis for the study and guided variable selection and analysis. The BHSU is widely used to examine health services use, including substance abuse services [14,18-20] and whether service utilization is equitable. According to this model, service utilization is equitable when services are fairly distributed on the basis of need rather than on the basis of non-need factors [18].

More specifically the BHSU suggests that health service utilization is a function of the separate and combined influence of three components: factors that *predispose *individuals to seek health care (e.g. demographic, attitudinal-belief variables), *enabling *conditions that allow the person to attain needed health services (e.g. awareness of services, affordability, psychological and cognitive factors), and the *need *for these services (or perceptions that problem levels are severe enough to warrant the use of health services) [14,18-20]. The model also suggests a causal ordering of these three components. Specifically, service need factors are considered the most important, proximal determinants of service use; predisposing factors are relatively weak influences on use; and enabling/restricting factors mediate the influence of treatment need on service access [18-20].

Using this framework, this study aimed to (i) explore whether access to substance abuse treatment is equitable and (ii) identify the profile of variables associated with treatment utilization (and non-utilization) for people from disadvantaged communities in Cape Town, South Africa.

## Methods

### Study design

This study used a case-control design to compare cases and controls on variables thought to be associated with substance abuse treatment utilization. This study defined cases as persons from disadvantaged communities with substance abuse problems who reported substance abuse treatment utilization in the 12 months preceding the study ("treatment use"). Controls were defined as persons from disadvantaged communities who had not used substance abuse treatment prior to this study, despite having substance abuse problems that required treatment ("no use").

To be eligible for participation in this study, potential cases and controls had to be at least 18 years old, live in one of the study's target communities, earn less than ZAR 2500 per month (at the time of the study 1 USD was the equivalent of ZAR 9.00); have substance abuse problems (either treated or untreated); and provide consent to participate in the study. In addition, controls had to report never having accessed substance abuse treatment services, despite having an objective need for such services. For potential controls, we used the Texas Christian University (TCU) Drug Screen to objectively assess the need for treatment [21]. For this screen, a composite score of 3 or greater indicates relatively severe drug-related problems that correspond to a DSM-IV-TR drug dependence diagnosis.

To control for selection bias, we used frequency matching techniques to match cases and controls on gender and race dimensions. In an attempt to limit recall bias that may have arisen from the collection of retrospective data, we used a time-line follow back (TLFB) procedure to assist with the collection of retrospective data on alcohol and drug use. Specifically, the TLFB procedure uses a calendar to assist the participant to reconstruct their daily patterns of alcohol and other drug use for a given period of time [22]. This procedure is widely used to quantify alcohol and drug use as an outcome measure for treatment research. Not only is this method comparable to other methods of reconstructing prior alcohol and drug use patterns but has also been proven to improve the accuracy of retrospective data collection [22,23].

### Recruitment and data collection procedures

From June 2006 through January 2007, we recruited convenience samples of participants using non-random techniques. Cases ("treatment use") were recruited using purposive sampling techniques. First, cases were identified at nonprofit substance abuse treatment facilities in the Cape Town metropole. These facilities were identified as starting points for sampling as they offer free or low-cost services and therefore are the most likely to serve clients from poor communities. Counsellors from these facilities were trained to screen recipients of services for study eligibility. Of the 440 persons screened, all met the study's inclusion criteria. Having established eligibility, counsellors obtained written consent to gather locator information from the recruits and pass this contact information on to the study's fieldworkers. Fieldworkers then contacted these recruits to obtain written informed consent to conduct an interview. Only six recruits refused to participate in the interview. This interview took approximately 90 minutes to complete.

Controls ("non-use" group) were recruited by a team of experienced fieldworkers using nonrandom snowball sampling techniques. To ensure controls represented the population of persons with substance abuse problems in disadvantaged communities, subjects were recruited from a broad range of these communities representing each of the six districts in the Cape Town metropole. More specifically, two communities from each district were purposively selected as target communities for sampling. To be selected for sampling, the community had to meet the following criteria: consistently appear in the South African Community Epidemiology Network on Drug Use's list of top ten residential areas for substance-related problems; have been classified as a "Black African" or "Coloured" residential area under the apartheid regime; have high levels of health and social problems; and be a low-income area.

Fieldworkers entered these communities by contacting community organizations, leaders, and members with known interests in the substance abuse field and asking them to identify potential recruits. Key informants were assured that their anonymity would be protected. Key informants were easily able to identify controls. In part, this is due to the social structure of poor communities in South Africa, where people live in close confines and share resources. In these communities, privacy is rare and keeping problems hidden is often difficult. Fieldworkers then contacted potential controls (who served as seeds or starting points for snowball sampling) to obtain consent to screen them for study eligibility. Of the 559 potential controls screened, only four did not meet the study's eligibility criteria; primarily because they scored below three on the TCU drug screen. For eligible participants, fieldworkers obtained written consent to conduct a full interview. Fieldworkers provided participants with refreshments, feedback, and referrals to substance abuse services where requested. No other incentives were provided to participants. Ethical approval for this study was granted by the Ethics Review Board of the Faculty of Humanities at the University of Cape Town.

### Participants

The final sample consisted of 434 cases and 555 controls (N = 989), representing an overall response rate of 98.3%. Several factors may have contributed to this high response rate. First, due to the nature of township life in Cape Town, drug problems are often not hidden in these communities, therefore drug users were neither surprised nor offended when approached by our team of fieldworkers. Second, our team of fieldworkers and the fieldwork manager were known to our target communities and all had been involved in community mobilization efforts. As such, they were easily able to gain the trust of the various community gatekeepers.

For the final sample, Chi-square tests of association revealed that cases and controls did not differ by gender or race. Similarly, independent sample *t *tests showed that the mean age and level of education was not significantly different for cases and controls (Table 1).

**Table 1:** Demographic information for the overall sample (N = 989)

Variable	Cases	Control	Chi-square/t -test (p)	Overall
**Male**	54.4% (236)	50.3% (279)	1.65 (0.20)	52.1% (515)
**Female**	45.6% (198)	49.7% (276)		47.9% (474)
**Black/African**	50.9% (221)	50.3% (279)	0.04 (0.84)	50.6% (500)
**Coloured**	49.1% (213)	49.7% (276)		49.4% (489)
**Mean age in years(SD)**	24.95 (4.81)	25.43 (5.98)	1.38 (0.17)	25.22 (5.51)
**Mean education - grade (SD)**	11.55 (1.57)	11.45 (1.52)	-0.95 (0.34)	11.50 (1.54)
**Total (N)**	434	555	-	989

### Measures

Our interview schedule included measures of substance abuse treatment use, need for treatment, factors thought to predispose individuals to accessing treatment, and factors thought to enable or restrict the use of services.

#### Use of substance abuse treatment

The criterion variable for this study was access to substance abuse treatment. This was assessed by the question: "Have you ever received treatment ("rehab") for an alcohol or drug problem?" This item had a "yes" (1) or a "no" (0) response.

#### Need for treatment

The questions: "Do you think you have an alcohol or drug problem?" and "Have other people suggested that you need help to change your use of alcohol or drugs?" examined internally and externally perceived treatment need, respectively. These items had a "yes" (1) or "no" (0) response. Perceived severity of drug use, an indicator of need for treatment, was assessed by the question, "How serious do you think your alcohol and drug use is?" with responses ranging from "Not at all serious" (1) to "Extremely serious" (4).

The 19-item Stages of Change, Readiness and Treatment Eagerness Scale (SOCRATES-8D) measured readiness to change substance use; a situational indicator of perceived need [24]. The SOCRATES consists of the 7-item problem recognition, 4-item ambivalence and the 8-item taking steps to change subscales. Higher scores on these subscales indicate greater readiness to change. This study obtained alpha coefficients ranging between .91 and .95 for the subscales.

#### Predisposing factors

Predisposing factors included age, gender, race/ethnicity, education level, and neighbourhood disadvantage, all of which have been associated with services utilization in the literature. Education level was based on the number of years of education received, treated as a continuous variable. The Neighbourhood Environment Scale (NES) measured neighbourhood disadvantage [16]. Specifically, items on this scale explore participants' perceptions about the extent to which there is crime, neighbourhood disrepair (broken windows, derelict buildings and refuse), drug availability and dealing, public drunkenness, social disobedience, damage to property, and poverty in their neighbourhood. For this study, the wording of the NES was adapted for an adult population and item responses were changed to range on a 5-point Likert scale from "strongly disagree" (1) to "strongly agree" (5). For this study, the adapted version had good internal consistency, with a Cronbach alpha coefficient of .82 being obtained.

#### Enabling and restricting factors

Enabling/restricting factors included logistic and psychological factors. Regarding the logistic factors, a 5-item "Affordability scale" was constructed to measure the extent to which treatment and transport costs act as barriers to access. Items were taken from Miller and Tonigan's (1995) "Barriers questionnaire" [25] and included items examining the extent to which respondents had the money to pay for treatment, to pay for transport to the nearest facility, medical insurance to pay for treatment, and were responsible for financially supporting others while in treatment. Items are rated on a 5-point Likert scale and responses are aggregated to give a composite score, with higher scores indicating more treatment and transport-related cost barriers. A Cronbach alpha coefficient of .84 was obtained for this scale. Participants were also asked whether competing financial priorities, specifically the need to pay for food and shelter, limited substance abuse treatment access. Responses for this item were coded as "yes" (1) or "no" (0).

Awareness of substance abuse treatment services was examined through asking participants to list all known treatment services. The number of known treatment facilities was then calculated, with larger numbers indicating greater awareness of services. The geographic accessibility of treatment was examined through asking participants to estimate the time it took (in 15 minute intervals) to travel to the nearest service. In addition, a 3-item "Delays in accessing treatment" scale was used to examine delays in accessing treatment due to distance, gatekeepers, and waiting lists. These items were taken from Miller and Tonigan's (1995) "Barriers questionnaire" [25] and included items on the extent to which whether respondents were placed on waiting lists to get into treatment, required reports from gatekeepers prior to accessing services, and lived far from the nearest treatment. Items are rated on a 5-point Likert scale and responses averaged to give a composite score, with higher scores indicating more delays in accessing treatment. A Cronbach alpha coefficient of .72 was obtained for this scale.

Psychological functioning was examined through the use of TCU's depression and anxiety scales. For these scales, higher scores indicate greater levels of depression and anxiety [26]. This study obtained Cronbach alpha coefficients ranging from .81 to .92 for these scales. In addition, we used the 10-item Stigma Consciousness Scale to examine internalized stigma. Items on this scale examine the extent to which respondents believe that others are prejudiced against them as drug users, they are not treated as equals, they are judged negatively on the basis of their drug use, and that people's negative judgements of their drug use affects them personally. Higher scores on this scale indicate greater expectations of being judged negatively due to one's substance use [15]. A Cronbach alpha coefficient of .84 was obtained for this scale.

Abstinence-specific social support was examined via the TCU social support scale. Item on this scale examine the extent to which respondents: have people close to them who motivate them to stay clean and sober, live in alcohol and drug free environments, have people close to them who respect their efforts in treatment, have good friends who do not use drugs, and have close family who help them stay away from drugs. Higher composite scores on this scale indicate greater levels of external support for treatment and abstinence [26]. This study obtained a Cronbach alpha coefficient of .77 for the scale.

Finally, a 10-item "Treatment concerns" scale was constructed to measure concerns about the substance abuse treatment process. Items on this scale examine fear of talking in groups, fear of revealing one's personal life, concerns about withdrawal, concerns about what might happen in treatment, and concerns about the kind of treatment that may be provided. These items were all taken from Miller and Tonigan's (1995) "Barriers questionnaire" [25]. For this scale, items are rated on a 5-point Likert scale and responses averaged to give a composite score, with higher scores reflecting greater concern about the treatment process. A Cronbach alpha coefficient of .90 was obtained for this scale.

### Analysis

Bivariate comparisons of the utilization variable and the predisposing, enabling and need measures were conducted. Specifically, Chi-square tests of association were conducted on categorical predisposing, enabling, and need-for-treatment variables by utilization and odds ratios were calculated to measure the strength of these associations. Independent sample *t*-tests were used to compare the utilization groups on continuously scaled predisposing, need-for-treatment and enabling variables. Following this, a hierarchical logistic regression procedure was performed with utilization as the dependent variable and the variables in the need-for-treatment, predisposing, and enabling/restricting variable domains significantly associated with utilization in bivariate analyses entered as block variables. This allowed us to explore the contribution that each variable domain made to the estimated variance in utilization, while controlling for the influence of the other variable domains. Inequity was determined by examining the significance and odds ratios of non-need relative to need-for-treatment variables. Gender and race were entered as covariates in Block 1. Following this, three regression models were evaluated in hierarchical fashion, beginning only with the need variables as predictors (Model 1) and culminating in a model that included need-for treatment, predisposing and enabling/restricting variable domains.

## Results

### Bivariate analyses

#### Predisposing variables

For the predisposing variables, the demographic variables of gender (χ^2 ^(1, N = 989) = 1.65, *p *= 0.199) and race (χ^2 ^(1, N = 989) = 0.04, *p *= 0.840) were not significantly associated with substance abuse treatment utilization (Table 2). The only predisposing variable significantly associated with treatment utilization was the continuous variable, the NES (Table 3). On this scale, controls reported greater neighbourhood disadvantage than cases. Age and level of education were not significantly associated with utilization (Table 3).

**Table 2:** Bivariate associations between categorical predisposing, need for treatment and enabling variables by utilization (N = 989)

Variable		Cases % (n)	Control (% (n)	Chi-square	***OR ***^***a ***^***(95% CI***^**b**^***)***
**Predisposing variable**					
**Gender**	Female	49.7 (276)	45.6 (198) 54.4 (236)	1.65	1.18 (0.92-1.52)
	Male	50.3 (279)			
**Race**	Black	50.3 (279)	50.9 (221)	0.04	0.97 (0.76-1.25)
	Coloured	49.7 (276)	49.1 (213)		

**Treatment need variables**					
**Others suggest treatment**	Yes	90.3 (392)	71.0 (394)	55.80(1)***	3.81 (2.64-5.51)
	No	9.7 (42)	29.0 (161)		
**Seriousness of use**	not at all serious	10.6 (46)	26.5 (147)	199.30 (4)***	
	Slightly serious	8.5 (37)	15.0 (83)		
	Moderately serious	6.9 (30)	26.3 (146)		
	Considerably serious	25.6 (111)	18.7 (104)		
	Extremely serious	48.4 (210)	26.3 (75)		

**Enabling/restricting variables**					
**Competing financial priorities**	Yes	40.1 (174)	73.5 (408)	112.33 (1)***	0.24 (0.18-0.31)
	No	59.9 (260)	26.5 (147)		

* α < .05; ** α < .01; *** α < .001

*α < .05; ** α < .01; *** α < .001;

^a ^Estimated odds ratio

^b ^95% confidence interval

**Table 3:** Independent sample *t *tests for continuous predisposing, need and enabling variables by utilization

Variables	No use (Controls) Mean (SD)	Treatment use (Cases) Mean (SD)	*t *value	df	Effect size (d)
**Predisposing variables**					
Age (years)	25.43 (5.98)	24.95 (4.81)	1.34	987	0.08
Level of education (years)	11.45 (1.52)	11.55 (1.57)	-0.95	987	0.06
Neighbourhood Environment scale	42.36 (3.43)	41.42 (5.07)	-7.93**	658	0.22

**Need variables**					
SOCRATES Problem Recognition	22.25 (7.15)	27.13 (5.57)	-11.70***	986	0.76
SOCRATES Ambivalence	13.31 (3.93)	15.56 (2.89)	-9.99***	975	0.65
SOCRATES Taking steps.	17.44 (4.65)	25.07 (8.24)	-18.39***	987	1.14

**Enabling variables**					
Affordability barriers	38.76 (6.23)	27.91 (9.46)	20.66***	854	1.39
Number of known treatment centres	1.06 (0.97)	4.00 (1.84)	-30.27***	619	2.07
Treatment concerns	26.43 (8.54)	29.70 (7.71)	-6.23***	987	0.40
Delays in treatment	37.63 (5.80)	31.90 (9.76)	10.83***	664	0.73
Time to treatment (minutes)	3.63 (0.58)	2.84 (0.80)	21.95***	769	1.46
Stigma consciousness	7.63 (1.53)	8.59 (1.64)	-9.44***	898	0.61
TCU Abstinence support	35.28 (5.56)	37.43 (4.66)	-6.62***	982	0.41
TCU Depression	32.51 (7.35)	38.31 (7.85)	-11.94***	987	0.77
TCU Anxiety	34.12 (8.66)	39.19 (7.90)	-9.61***	964	0.61

* α < .05; ** α < .01; *** α < .001

#### Need- for-treatment variables

The categorical variable, "others suggesting the need for AOD treatment" was significantly associated with utilization (χ^2 ^(1, N = 989) = 55.80, *p *< 0.001). The odds of utilizing treatment were three-fold greater for participants for whom a significant other had suggested the need for treatment compared to those who had not received this advice (OR = 3.81; CI (95): 2.64-5.51) (Table 2). Perceived seriousness of substance abuse problems also was significantly associated with utilization (χ^2 ^(4, N = 989) = 199.30, *p *< 0.001), with participants who reported "extremely serious drug problems" more likely to fall into the treatment use group compared than the treatment non-use group (Table 2).

Significant differences were found between cases and controls on the SOCRATES subscales. The treatment use group obtained significantly higher scores on the problem recognition, ambivalence and taking steps subscales than the treatment non-use group (Table 3).

#### Enabling/restricting variables

The variable "competing financial priorities" was significantly associated with treatment utilization (χ^2 ^(1, N = 989) = 112.33, *p *< 0.001). Participants with competing financial priorities had four-fold greater odds of not utilizing treatment than those without competing financial needs (OR = 0.24; CI (95): 0.18-0.31) (Table 2). The treatment non-use group also reported significantly less awareness of where to go for substance abuse treatment, longer travelling times to the nearest treatment centre, longer waiting periods and delays in accessing care, and more affordability barriers than the treatment use group (Table 3). For the psychological enabling/restricting variables, the treatment use group reported significantly more depression and anxiety, more stigma consciousness, higher levels of social support for treatment and abstinence, and more concerns about the process of treatment than the treatment no use group (Table 3).

### Hierarchical logistic regression

A hierarchical logistic regression of treatment utilization was conducted while controlling for the potentially confounding effects of race and gender. The addition of a block of need variables in Model One had substantially better predictive utility than the model with the intercept only (Δ*χ*^2 ^(8; N = 989) = 414.33, *p *< .001). This model accounted for approximately 46% of the estimated variance in access (Nagelkerke R^2 ^= .460). For this model, four need variables had significant partial effects on utilization: perceived seriousness of substance use (where those who reported extremely serious problems where significantly more likely to have utilised treatment services than those with problems that were "not at all serious"), others suggesting the need for treatment, and the SOCRATES ambivalence and taking steps subscales (Table 4). Despite these findings, Model One did not fit the data adequately (Hosmer-Lemeshow *χ*^2 ^(8; N= 989) = 15.56, *p *= 0.04).

**Table 4:** Summary of hierarchical logistic regression analysis using predisposing, enabling and need factors as predictors of substance abuse treatment utilization ^a, b^

Predictor variables	Model 1		Model 2		Model 3	
	
	*Wald (df)*	***OR ***^**c **^***(95% CI***^d^***)***	*Wald(df)*	***OR ***^**c **^***(95% CI***^**d**^***)***	*Wald(df)*	***OR ***^**c **^***(95% CI***^**d**^***)***
***Need for treatment***						
Seriousness of drug use: reference "not at all serious")						
Slightly serious	3.55(1)	0.55 (0.29-1.02)	3.34(1)	0.56 (0.30-1.04)	0.15(1)	1.36 (0.30-6.22)
Moderately serious	18.39(1)***	0.24 (0.13-0.46)	18.59(1)***	0.24 (0.12-0.45)	2.13(1)	0.38 (0.11-1.39)
Considerably serious	0.01(1)	1.01 (0.54-1.89)	0.02(1)	1.05 (0.56-1.97)	5.45(1)*	5.11 (1.30-20.09)
Extremely serious	10.87(1)**	2.92 (1.54-5.89)	10.67(1)**	2.92 (1.54-5.54)	8.79(1)**	8.51 (2.07-35.03)
Others suggest treatment need (yes)	14.12(1)***	2.36 (1.51-3.70)	13.83(1)***	2.35 (1.50-3.68)	1.97(1)	1.99 (0.76-5.22)
SOCRATES-problem recognition	3.03(1)	0.96 (0.92-1.01)	3.33(1)	0.96 (0.91-1.00)	2.99(1)	0.92 (0.83-1.01)
SOCRATES-ambivalence	9.33(1)**	1.13 (1.05-1.22)	10.01(1)**	1.14 (1.05-1.23)	20.39 (1)***	1.42(1.22-1.65)
SOCRATES-taking steps	105.02***	1.16 (1.13-1.19)	101.46(1)***	1.16 (1.13-1.19)	2.90(1)	1.06 (0.99-1.13)

***Predisposing variables***						
NES	-	-	5.18(1)*	0.95 (0.91-0.99)	0.11(1)	0.99 (0.90-1.08)

***Enabling variables***						
Number of known treatment centres	-	-	-	-	72.33(1)***	5.80 (3.85-8.75)
Travelling time to treatment	-	-	-	-	64.35(1)***	0.07 (0.04-0.14)
Competing financial priorities (No)	-	-	-	-	20.23(1)***	5.31 (2.56-10.98)
Affordability barriers	-	-	-	-	30.02(1)***	0.85 (0.81-0.91)
Delays in accessing treatment	-	-	-	-	7.16(1)**	0.93 (0.88-0.98)
TCU depression	-	-	-	-	1.61(1)	1.05 (0.97-1.14)
TCU anxiety	-	-	-	-	3.35(1)	0.93 (0.86-1.01)
Abstinence support	-	-	-	-	4.66(1)*	0.94 (0.87-0.99)
Treatment concerns					32.08 (1)***	1.17 (1.11-1.23)
Stigma consciousness					0.45(1)	1.08 (0.86-1.34)

*α < .05; ** α < .01; *** α < .001;

^a ^Controlling for confounding effects of gender and race

^b ^Blank spaces indicate that the variable did not enter the equation

^c ^Estimated odds ratio

^d ^95% confidence interval based on the Wald's test

The addition of a block of predisposing variables in Model Two increased the predictive value of the model (χ^2^(1; N = 989) = 5.33, *p *< .05) by approximately 1%. Model Two was able to predict an estimated 47% of the variance (Nagelkerke R^2 ^= .465). However the general fit of the model to the data remained inadequate (Hosmer-Lemeshow χ^2 ^(8; N= 989) = 20.34, *p *= 0.009). For this model, the only predisposing variable that was significantly (albeit weakly) associated with utilization was neighbourhood environment. All the need-for-treatment variables included in Model One remained significantly associated with utilization in Model Two, with the strength of these associations being unchanged (Table 4).

In Model Three, an enabling/restricting variable block was added to the model while controlling for the influence of the other domains. This model had substantially better predictive utility than the model with the intercept only (χ^2 ^(10; N = 989) = 663.96, *p *< .001). The addition of this enabling variable block increased the amount of variance accounted for by approximately 43%, with the final model predicting about 89% of the estimated variance (Nagelkerke R^2 ^= .898). Furthermore, with the addition of this block of variables, the general fit of the model to the data became adequate (Hosmer-Lemeshow χ^2 ^(8; N= 989) = 0.71, *p *= 1.000).

NES (a predisposing variable) and several of the need-for- treatment variables associated with utilization in Models One and Two were no longer associated with utilization in Model Three (Table 4). For this final model, "Perceived seriousness of drug use" was one need variable that had significant partial effects on utilization. Compared to participants who reported that their drug use was "not at all serious", participants who reported that their drug use was "considerably serious" were five times more likely to utilize treatment and participants who reported their problems were "extremely serious" were eight times more likely to enter treatment when the influence of other variables was controlled for. The SOCRATES ambivalence scale was the only other need variable significantly (albeit weakly) associated with utilization (Table 4).

In contrast, significant and strong associations were found between utilization and several of the logistic enabling/restricting variables. Awareness of services was positively associated with utilization. For every additional treatment centre that a participant knew of, the odds of utilizing treatment increased by a multiplicative factor of 5.81. Geographic accessibility was negatively associated with utilization. For every 15 minute increase in travelling time to treatment, the odds of not utilizing treatment services increased by a multiplicative factor of 14.29. Affordability barriers and competing financial priorities also were significant partial predictors of utilization. Participants without competing financial priorities had roughly a five times greater likelihood of utilizing treatment compared to their respective counterparts with competing financial priorities (Table 4). In addition, every one-unit increase in the affordability barriers scale augmented the odds of not accessing treatment by a multiplicative factor of 1.2 (OR = 1.18; CI (95): 1.10-1.24). Although significant, the effects found for the delays in accessing treatment scale were weak (Table 3). In contrast, few psychological enabling/restricting variables were associated with treatment service utilization. Only the abstinence support scales and the treatment concerns scales were significantly, albeit weakly, associated with utilization (Table 4).

## Discussion

Contrary to findings from studies conducted in developed countries where need for treatment variables are the principal determinants of substance abuse treatment utilization [9,10], our findings show that treatment need and non-need variable domains account for almost equal proportions of the estimated variance in service utilization and therefore are equally strong determinants of service utilization. Given that equity in service utilization is thought to exist where need for treatment (rather than non-need) factors are the principal determinants of treatment access [18], our findings point to inequities in current patterns of substance abuse treatment utilization among disadvantaged communities in Cape Town.

The important effect that enabling/restricting variables have on the chances of substance abuse treatment utilization also is evident from the fact that the addition of an enabling/restricting variable block to our multivariate model of utilization significantly improved the model's fit and weakened the influence of several predisposing and need for treatment variables on utilization. These results suggest that while treatment need is necessary for substance abuse service utilization to occur, multiple barrier variables within disadvantaged communities in Cape Town attenuate the influence of treatment need on service utilization. To improve the uptake of substance abuse treatment services for people from poor communities in Cape Town, it is thus vital that the barriers to treatment use are reduced.

More specifically, our study identified financial and geographic access barriers to substance abuse treatment use for people from poor communities in Cape Town. In terms of the financial barriers, greater concerns about the affordability of treatment and the presence of competing financial priorities diminished the chances of substance abuse treatment utilization, even after controlling for the influence of predisposing and treatment need variables. However, treatment affordability concerns were less of a barrier to service use than competing financial priorities related to the provision of food and shelter. This is probably because free and low cost treatment options are available in the Cape Town region, even though the availability of these services is limited [6]. In contrast, the presence of competing financial priorities related to survival needs was a very strong barrier to treatment utilization. The saliency of this barrier is not surprising given the widespread poverty and unemployment present in disadvantaged communities [8]. In such contexts, priority is understandably first given to the meeting of survival needs rather than the need for what is viewed as non-essential health services [27].

The vulnerability of people from disadvantaged communities to financial barriers may also account for the finding that geographic access barriers (especially lengthy travel times to treatment) significantly impact on the likelihood of substance abuse treatment utilization. While the reason for the strong negative association between travel time to treatment and service utilization was not investigated here, it is quite plausible that the high costs associated with South African public transport and the potential loss of income associated with difficult and lengthy commutes may make lengthy travel times unaffordable to South Africans from poor communities with competing financial priorities and low incomes. This claim is supported by findings from previous studies which suggest that financial barriers often compound geographic access barriers [28].

Regardless of the reasons for these findings, it is clear that improvement in treatment uptake among people from disadvantaged communities will occur only when these geographic access and financial barriers are reduced. One strategy for diminishing these barriers is to introduce mobile outpatient treatment services into disadvantaged communities. Not only would such services improve treatment availability, but they would also reduce travel time to treatment and the overhead costs associated with facility-based treatment [29]. In Cape Town, large mobile vans are already used to provide primary health and education services and are well accepted by poor communities. There is no foreseeable reason why similar vans could not be used to provide substance-related treatment services. Another strategy for reducing these barriers would be for service providers to provide prospective clients from disadvantaged communities with tokens for public transport or transport services. Similarly, for prospective clients who express concern about their ability to meet their basic needs while in treatment, the provision of food vouchers may provide some financial relief. When used as part of contingency management, the provision of these vouchers may have the added benefit of encouraging treatment engagement and positive outcomes [30].

Apart from these barriers, this study also found that awareness of substance abuse treatment services is an important enabling resource for people from disadvantaged communities, with greater awareness of available substance abuse treatment facilities increasing the likelihood of treatment utilization. Although it is hard to unpack whether participants who had accessed treatment had better awareness of services prior to or as because of their treatment experiences, we did attempt to limit recall bias by using timeline follow-back techniques [22]. Also, it should be noted that awareness of where to go for substance abuse treatment was very low among people who had never accessed services, with participants on average only being able to name one treatment facility. This may be due to the fact that current drug awareness programmes are based in settings that are not frequented by drug users (e.g. health promotion events at schools and in primary health clinics). Given that awareness of where to go for treatment is an important condition for help-seeking [9], limited awareness of where to go for treatment within disadvantaged communities needs to be addressed as a matter of urgency. One way of improving awareness would be to introduce community-based outreach programmes that actively seek to improve awareness of services among current drug users (rather than among non- users). Through training outreach workers to detect alcohol and drug problems, these workers can also be used as resource for screening and referral to existing treatment services.

Apart from these enabling/restricting variables, this study also found that perceived severity of drug use, a treatment need variable, was a strong predictor of treatment utilization. Persons who self-reported that they had considerable or extremely serious problems associated with their drug use were significantly more likely to access treatment than those with less serious problems. While people with more severe drug problems should be the most likely to access services, nevertheless it is concerning that people who self-report moderate problems related to their drug use are no more likely to receive treatment services than people who report few problems related to their drug use. As treatment access only seems to occur when drug problems are associated with considerable harms, opportunities for intervening before problems become severe and intractable are lost. In a context where resources for treatment are scarce, policy makers and service planners need to give serious consideration to how best to distribute treatment monies. In the long-term, expanding existing services through the addition of low intensity services aimed at people with moderate problems may have a more favourable cost-benefit ratio than the addition of more high intensity (and high cost) services.

Findings from this study should be considered in the light of several limitations. First, the study's case-control design prohibits a temporal examination of the factors associated with treatment utilization and therefore we cannot comment on the directionality or causality of these relationships. Second, our rather simple, dichotomous definition of utilization did not allow us to examine nuances within the broad categories of "treatment use" and "treatment non-use." It is quite possible that individuals who had needed but had never tried to access treatment may have differed qualitatively from individuals who had unsuccessfully tried to access needed services in the past. In addition, within the treatment use group, utilization may have varied along a frequency continuum, with participants with little (but some) contact with treatment services varying in potentially important ways from participants with more frequent treatment service utilization. Third, our definition of cases as "individuals who had utilised treatment in the 12 months preceding the study" precluded persons who may have utilised services prior to this period. While these individuals may have contributed to a more in-depth understanding of access, we chose this strict definition of cases to limit the possibility of recall bias that may have resulted from fallible memory of events preceding 12 months. Fourth, our strict selection criteria may have reduced the variability of the sample and contributed to most predisposing variables not being associated with treatment utilization. Fifth, while the data from this study are now four years old, it is quite unlikely that major changes have occurred. Given that the uptake of treatment services has contracted in recent years [7], it is quite possible that the barriers we identify here are even more entrenched. In addition as the sample was limited to adults from the Western Cape, the extent to which findings are relevant for adolescents or persons from more rural or other urban regions in South Africa is questionable. Yet, compared to the other provinces, the Western Cape is better resourced in terms of substance abuse treatment services. For example, several of the more rural provinces only have one substance abuse treatment service [7]. For that reason, the structural and population-level barriers to treatment utilization we describe here are quite likely even more salient in other provinces. Finally, as the study was limited to Black/African and Coloured adults and excluded White South Africans, it is impossible to determine whether access to substance abuse treatment is equitably distributed across the various race and ethnic groups in the region. However, the question of whether treatment services are equitably distributed is important and warrants further investigation because of the potentially important implications findings have for treatment policy and resource allocation.

These limitations draw attention to the need for additional research on substance abuse treatment utilization in South Africa. Specifically, longitudinal prospective studies that track people with substance use disorders over time and allow researchers to unpack the temporal associations between predisposing, treatment need, enabling/restricting variables and substance abuse treatment utilization are needed. In addition, these longitudinal studies will allow researchers to distinguish between people who need treatment but do not try to access services, people with failed attempts to access substance abuse treatment, and people who succeed in utilizing treatment services when needed. Secondly, concerns about the external validity of our findings highlight the need for studies on substance abuse treatment utilization in other parts of the country (particularly under-serviced rural regions) and for other population subgroups (such as adolescents). Thirdly, researchers should consider conducting experimental studies to test whether interventions to remove the structural barriers we identified significantly improve substance abuse treatment utilization for people from disadvantaged communities.

## Conclusions

This study is the first of its kind to examine factors associated with substance abuse treatment utilization in an African country. Despite some limitations this study provides good evidence that the use of substance abuse treatment services for people from poor communities in Cape Town is inequitable; with non-need factors being powerful predictors of substance abuse treatment utilization, especially financial, geographic access and awareness barriers. While need for treatment still plays a role in informing the likelihood of service use, only persons with the most severe problems utilize existing services. Taken together, these findings suggest the need for targeted interventions that address barriers to treatment utilization. Various strategies can be used to improve the uptake of treatment services by people from disadvantaged communities. For service planners, new services should be placed in locations easily accessible by public transport and in communities with high service needs and poor service coverage. Related to this, service planners should consider expanding the existing repertoire of services provided to include lower intensity early intervention services aimed at people with low to moderately severe drug problems. Third, outpatient mobile clinics offer a low cost and geographically accessible means of providing substance abuse treatment services, particularly as these clinics can be moved between and within communities. Fourth, financial and transport barriers can be addressed by providing food and transport vouchers to prospective clients as part of a contingency management strategy. This would have the added advantage of improving treatment adherence and outcomes. Finally, community-based outreach workers may be a useful resource for improving awareness of substance abuse treatment services within disadvantaged communities by educating people about when, where and how to access substance abuse treatment.

## Competing interests

The authors declare that they have no competing interests.

## Authors' contributions

BM conceived the study, participated in its design and coordination, and helped draft the manuscript; JL participated in the design and coordination of the study and helped draft the manuscript, SP helped draft the manuscript. All authors read and approved the final manuscript.
